# The role of novel programmed cell death in head and neck squamous cell carcinoma: from mechanisms to potential therapies

**DOI:** 10.3389/fphar.2023.1228985

**Published:** 2023-09-25

**Authors:** Yujie Xi, Ling Gao, Shaming Li, Kai Sun, Peishen Chen, Zhen Cai, Wenhao Ren, Keqian Zhi

**Affiliations:** ^1^ Department of Oral and Maxillofacial Reconstruction, The Affiliated Hospital of Qingdao University, Qingdao, China; ^2^ Key Lab of Oral Clinical Medicine, The Affiliated Hospital of Qingdao University, Qingdao, China; ^3^ Experimental Research Centre, China Academy of Chinese Medical Science, Beijing, China; ^4^ School of Stomatology of Qingdao University, Qingdao, China; ^5^ Department of Stomatology, People’s Hospital of Juxian, Rizhao, China; ^6^ Department of Stomatology, Linyi People’s Hospital, Linyi, Shandong, China

**Keywords:** head and neck squamous cell carcinoma (HNSCC), programmed cell death (CDDPCD), mechanism, natural products, potential therapies

## Abstract

Head and neck squamous cell carcinoma (HNSCC) is a common oral cancer with poor prognosis and for which no targeted therapeutic strategies are currently available. Accumulating evidence has demonstrated that programmed cell death (PCD) is essential in the development of HNSCC as a second messenger. PCD can be categorized into numerous different subroutines: in addition to the two well-known types of apoptosis and autophagy, novel forms of programmed cell death (e.g., necroptosis, pyroptosis, ferroptosis, and NETosis) also serve as key alternatives in tumorigenesis. Cancer cells are not able to avoid all types of cell death simultaneously, since different cell death subroutines follow different regulatory pathways. Herein, we summarize the roles of novel programmed cell death in tumorigenesis and present our interpretations of the molecular mechanisms with a view to the development of further potential therapies.

## Graphical Abstract

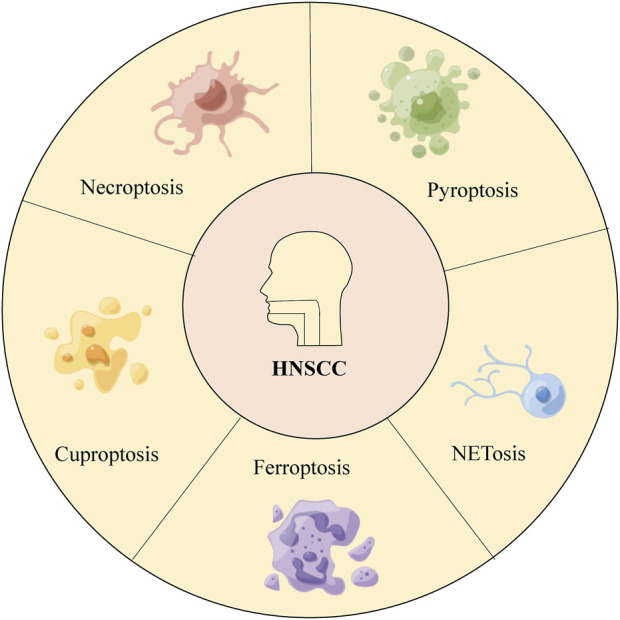

## 1 Introduction

Head and neck squamous cell carcinoma (HNSCC) is a collection of heterogeneous malignancies that occur in the mucosal surfaces of the oral cavity, paranasal sinuses, nasal cavity, salivary glands, pharynx, and larynx (Johnson et al., 2020). Currently, HNSCC ranks as the sixth most prevalent type of cancer worldwide, with over 930,000 new cases and 460,000 deaths in 2020 (Bray et al., 2018; Ferlay et al., 2019). Open surgery has been a traditional treatment for HNSCC, often followed by radiation or chemotherapy (Mehanna et al., 2010; Johnson et al., 2020). However, the limited effectiveness of these approaches in improving the unfavorable prognosis of patients can be attributed to the existence of numerous genetic and epigenetic alterations that underlie the molecular mechanisms involved. Frequently, the culprit is the complex signaling network involved in programmed cell death.

Three major risk factors for developing HNSCC—tobacco, alcohol, and HPV—have been frequently identified in the trial. These have also been found to be closely associated with the emergence of programmed cell death and several important drivers of this (Saad et al., 2015; Batta and Pandey, 2019; Ferris et al., 2021). In particular, forms of cell death targeting the PD1 receptor or its ligand PD-L1 are the focus in the case of HPV-positive HNSCC, indicating the probable direction of development for treatments in relation to each subtype of PCD. In a preclinical model of HNSCC, it has been shown that the combined cIAP/XIAP inhibition of ASTX660 and necrosis-mediated TP53 expression have a direct antitumor effect on HPV(+) subtypes (Xiao et al., 2019). The expression of a pyroptosis-associated gene index (NLRP1) and an apoptosis-related gene index (CDKN2A) has been found to predict better survival based on cell death index scores in two HPV-negative HNSCC cell lines and greater potential efficacy for HNSCC prognosis (Nan et al., 2023). This important finding further emphasizes the fact that ferroptosis-associated targeting of expression levels of the cystine transporter SLC7A11 are lower in HPV-positive (HPV + ve) tumors than in HPV-negative (HPV-ve) HNSCC, which has been observed using RNAseq data (both whole-tumor and single-cell sequencing) (Hémon et al., 2020). Thus, in the present day, we are eager to examine the deep pathogenesis of programmed cell death in HNSCC and seek effective treatments.

Cell death has been divided into accidental cell death (ACD) and regulated cell death (RCD) by the Nomenclature Committee on Cell Death (Galluzzi et al., 2018). RCD, also known as PCD, involves tightly structured signaling cascades, homeostasis, and pathological processes in multicellular organisms (Koren and Fuchs, 2021). Although a growing number of classical forms of PCD, such as apoptosis and autophagy, have been extensively studied and their significance now interpreted in various human pathologies, changes in the molecular mechanisms that regulate programmed necrosis are not commonly observed in cancer (Glick et al., 2010; Carneiro and El-Deiry, 2020). Various novel forms of programmed cell death, such as necroptosis, pyroptosis, ferroptosis, NETosis, and cuproptosis, play significant roles in the development, progression, and regression of HNSCC (Lee et al., 2018b). Thus, understanding novel PCD is a promising strategy to subvert treatment resistance, and may be of equal importance to an understanding of apoptosis. Therefore, it is necessary to determine the mechanisms underlying these types of PCD, as well as their connections to cancer.

**FIGURE 1:**
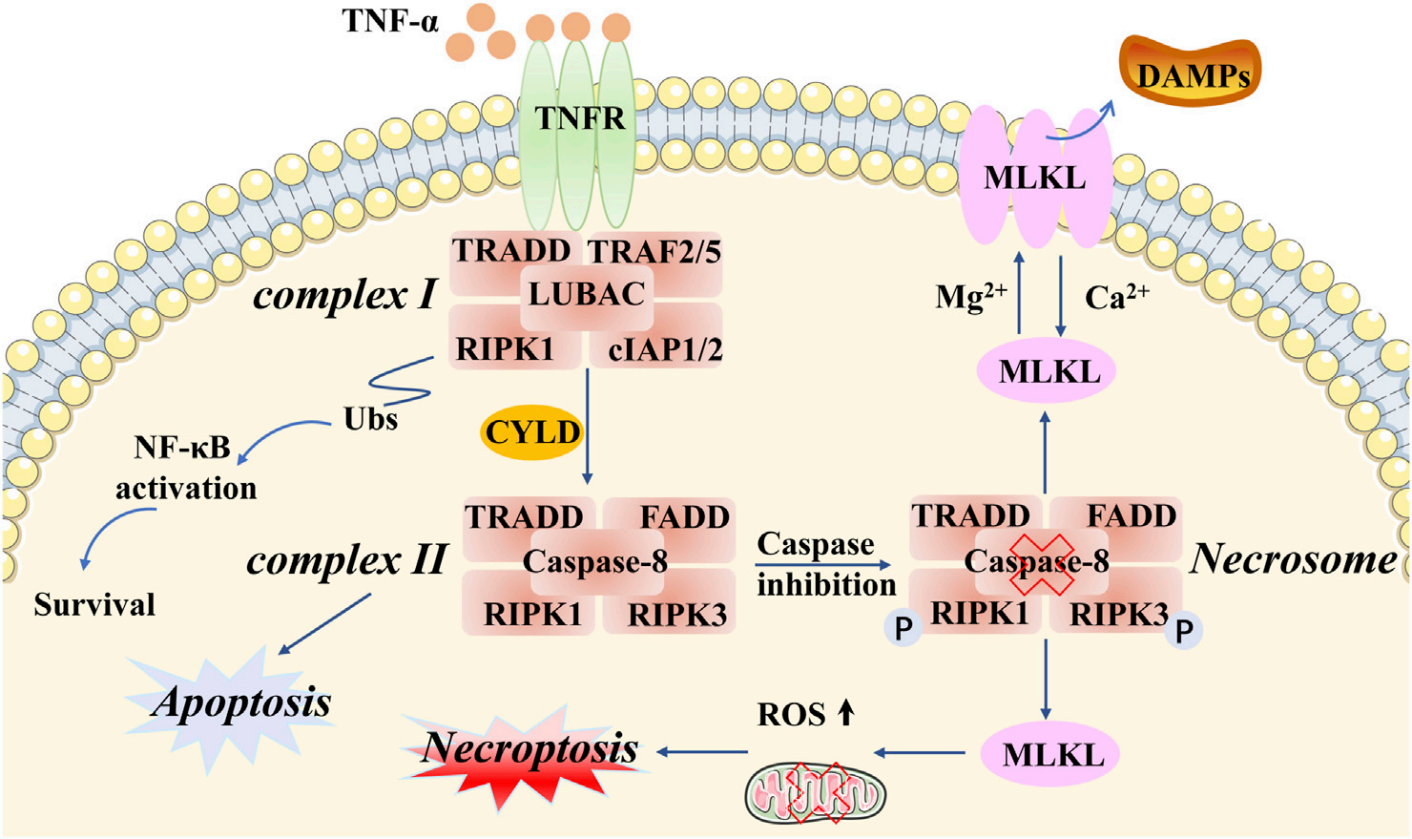
Molecular mechanisms of necroptosis. Necroptotic cell death can be induced by different types of stimuli and related receptors (e.g., TNF-α and TNFR). Particular attention can be paid to complex I and complex II. TRADD, RIPK1, TRAF2/5, and cIAP1/2 are recruited in complex I, which activates the protein RIPK1 and RIPK3. Complex II involves TRADD, RIPK1, FADD, RIPK3, and caspase-8. An important component of intracellular signaling is the formation of the necrosome in necroptosis, accompanied by caspase-8 inactivation and hyperphosphorylation of RIPK1/3. MLKL is a key downstream regulator of this pathway, exerting complex effects through multiple phosphorylation and ubiquitination events that determine the release of inflammatory DAMPs and ROS production, as well as mitochondrial division.

In this review, we shed light on the antitumor effects of nonapoptotic PCD in HNSCC and the molecular mechanisms targeting different PCD subroutines. Furthermore, we also discuss pathways and biomarkers for the use of natural products in relation to PCD and predict future orientations for clinical therapies (Figure 1).

## 2 Overview of mechanical networks in novel programmed cell death

Necrosis, previously known as the passive and unregulated cell suicide process, has been recognized as arising from irreversible damage to cells resulting from pathological processes (hypoxia; physical, chemical, and biological processes; or immune responses) (Degterev et al., 2005; Quail and Joyce, 2013).

Cells undergoing necrosis frequently exhibit damage to the plasma membrane, mitochondrial swelling, ATP depletion in the cell, and even the release of proinflammatory molecules (Edinger and Thompson, 2004). In fact, over the past decade, there has been growing recognition that a significant proportion of necrosis, actively mediated by the doomed cell, might in fact be a programmed and deliberate process. Hence, a comprehensive understanding of the different modes of programmed cell death is needed. An overview of key PCD modalities, namely, necroptosis, pyroptosis, ferroptosis, NETosis, and cuproptosis, has come into focus (Lee et al., 2018b; Zhu and Sun, 2018).

### 2.1 Necroptosis

Of the various types of novel PCD, necroptosis is the most extensively described and investigated. Necroptosis, showing a distinct mechanism from apoptosis in terms of its influence on mouse ischemic brain injury and neuroprotection, was first named by Professor Yuan of Harvard Medical School in 2005 (Degterev et al., 2005). This programmed form of necrosis features similar morphological changes to necrosis, including damage to the plasma membrane, lysis of endoplasmic reticulum, mitochondrial swelling, release of intracellular contents, production of proinflammatory cytokines, and even the elicitation of an immunologic response (Linkermann and Green, 2014; Tonnus et al., 2019; Yuan et al., 2019). The distinction from necrosis is that necroptosis strictly follows the activation of intracellular signaling pathways in cancer and actively consumes energy (Conrad et al., 2016; Peng et al., 2022). Notably, different types of stimuli can trigger necroptosis, such as lipopolysaccharide (LPS), interferon-γ (IFN-γ), Z-DNA-binding protein 1 (ZBP1), tumor necrosis factor alpha (TNF-α), death receptors (e.g., FAS), and toll-like receptors (e.g., TLR3 and TLR4) (Kaiser et al., 2013; Chan et al., 2015; Pasparakis and Vandenabeele, 2015; Newton et al., 2016). Let us take the most intensive study of tumor necrosis factor receptor 1 (TNFR1) as an example. Specifically, after TNF-α binds to TNFR1 on the plasma membrane, several downstream protein molecules are recruited into a form referred to as complex I, which consists of TNFR-associated death domain (TRADD), receptor-interacting protein kinase 1 (RIPK1), TNFR-associated factor 2 (TRAF2), TNFR-associated factor 5 (TRAF5), and E3 ubiquitin ligases cellular inhibitor of apoptosis 1 (cIAP1) linear ubiquitin chain assembly complex (LUBAC) (cIAP2) (Micheau and Tschopp, 2003; Bellomaria et al., 2010; Dondelinger et al., 2016). Following this, cIAPs function as E3 ligases that induce Lys63-domain polyubiquitination of RIPK1, as well as activating the nuclear transcription factor–kappa beta (NF-kB) pathway (Dondelinger et al., 2015; Annibaldi et al., 2018). Subsequently, with the help of deubiquitinases (e.g., CYLD), RIPK1 undergoes deubiquitination events to generate a new protein molecule form, including RIPK1, RIPK3, FADD, TRADD, and caspase-8, called the necrosome (also known as complex II). It appears that caspase-8 plays a critical role in this process. When caspase-8 is activated, it will inhibit phosphorylation in RIPK1 and RIPK3, and the inactivation of the necrosome leads to apoptosis. However, if caspase-8 is inactivated, then RIPK1 and RIPK3 will hyperphosphorylate, resulting in the activation of the necrosome and leading to necroptosis (Wang et al., 2008; Feoktistova et al., 2011; Lafont et al., 2018). Subsequently, the mixed-lineage kinase domain-like pseudokinase (MLKL) will be recruited by the activated necrosome, which causes two distinct checkpoints to be set to show biochemical features in the following steps. On the one hand, ectopic MLKL translocating towards the plasma membrane leads to the release of damage-associated molecular patterns (DAMPs) and an inflammation response (Rickard et al., 2014; Murai et al., 2018). On the other hand, MLKL activates DRP1 in the mitochondria via PGAM5, causing the accumulation of ROS and mitochondrial division (Wang et al., 2012; Remijsen et al., 2014; Basit et al., 2017; Liu et al., 2021b) (Figure 1). In brief, necroptosis is characterized by the pathogenically blocked catalytic activity of caspases and necrosome formation. However, it is also necessary to further study the relationship between cell necroptosis and tumorigenesis.

### 2.2 Pyroptosis

**FIGURE 2:**
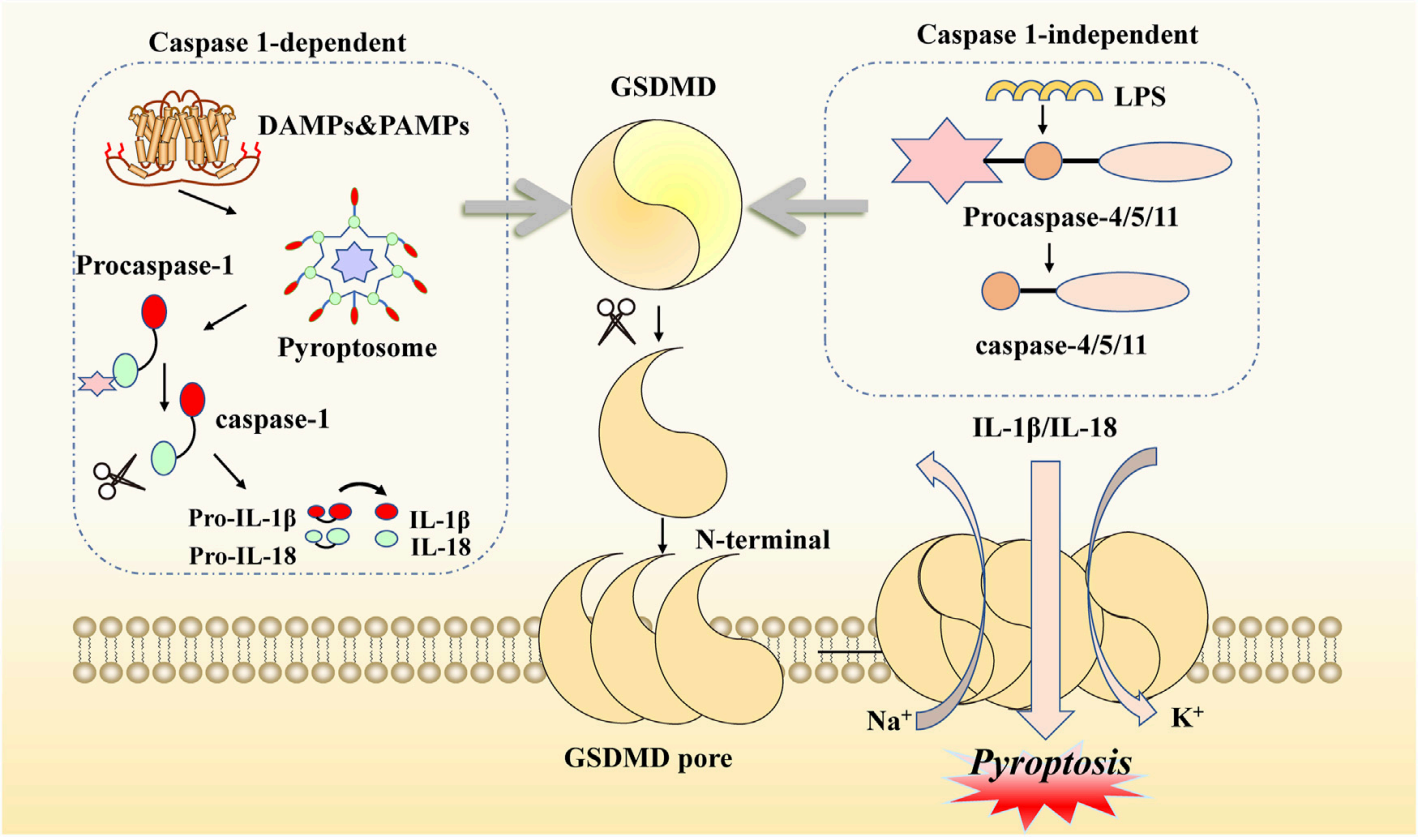
Molecular mechanisms of pyroptosis. This kind of cell death can be induced by two main pathways. When DAMPs and PAMPs activate the canonical pathway, NLRP3, ASC, and procaspase-1 are recruited as pyroptosomes, then hydrolyzed to form caspase-1. When LPS enters the organism, it can be activated to another pathway binding to procaspase4/5/11. Both of these need to be attached to GSDMD cleavage and produce GSDME N-fragments. The N-fragments of GSDME form cell membrane pores, resulting in pyroptosis. With the continuous accumulation of N-terminal formation of pores on the membrane, the extracellular transport of inflammatory factors (IL-1β/IL-18) occurs, as well as potassium ion outflow and sodium ion inward flow. Activated caspase-1 also promotes pro-inflammatory interleukins IL-1β and IL-18.

Pyroptosis is another form of novel PCD that is morphologically and biochemically distinct from apoptosis. Sperandio et al. discovered this inflammatory death phenotype first, observing overexpressed insulin-like growth factor 1 receptor (IGFIR) in a 293T cell line in 2000 (Sperandio et al., 2004). Compared with necroptosis, the typical features of pyroptosis are fewer cells with mitochondrial swelling, extensive cytoplasmic vacuolization with pyroptosome formation, pore formation, and rupture of the plasma membrane (Xu et al., 2021). Concerning molecular mechanisms, there are two main pathways mediating pyroptosis: one is a canonical inflammasome pathway, which is caspase-1-dependent, and the other is a non-canonical inflammasome pathway, which is caspase-1-independent (Fontana et al., 2020; Lu et al., 2022). Specifically, the former pathway is initiated by pathogen-associated molecular patterns (PAMPs) or damage-associated molecular patterns (DAMPs) via activation of pyroptosome signaling. This process can be divided into steps involving various types of stimuli, including nod-like receptors (NLRs) and absent in melanoma-like receptors (ALRs) (He et al., 2020; Li et al., 2022d). Taking the NLR family as an example: after recognition of specific pattern recognition receptors coupled with stimuli, the pyroptosome is assembled by NOD-like receptor thermal protein domain-associated protein 3 (NLRP3), apoptosis-associated speck-like protein containing a CRAD (ASC) complex, and pro-caspase-1 (Cookson and Brennan, 2001; Ramirez et al., 2018). Subsequently, procaspase-1 is converted to caspase-1, which hydrolyzes and processes the pro-inflammatory interleukins pro-IL1B and pro-IL18 into mature IL1B and IL18, respectively, leading to cleavage of gasdermin E (GSDME) (Miao et al., 2010; Rao et al., 2022). Meanwhile, the latter pathway is based on LPS in the cytoplasm of macrophages, monocytes, or other cells, directly binding caspase-4/5/11 to activate GSDMD. Therefore, GSDMD is a crucial participant in both the canonical and the non-canonical pathways of pyroptosis. GSDMD can be cleaved by the N-terminal and C-terminal junction domains of gasdermins, and activated N-terminal regions form pores in the cell membrane, resulting in the release of many pro-inflammatory actors, potassium efflux, and calcium influx (Lee et al., 2018a; Orning et al., 2019; Wang et al., 2020; Burdette et al., 2021). In addition, caspase-8 and caspase-3, which exert their effects in the upstream signaling of the apoptotic pathway, can also initiate pyroptosis via GSDME cleavage (Wang et al., 2017; Chen et al., 2019; Wu et al., 2022a). All these signals, including cell lysis, the release of cytoplasmic contents, and nuclear condensation, lead to a pyroptosis microenvironment (Quail and Joyce, 2013; Xia et al., 2019). Thus, this type of novel PCD elicits two main reactions that can lead to cell lysis, the release of cytoplasmic contents, nuclear condensation, and even damage to normal cells (Ding et al., 2016; Liu et al., 2016). Given the activation of all these inflammatory signals, the association of pyroptosis with innate immunity and disease needs to be considered (Figure 2).

### 2.3 Ferroptosis

Ferroptosis, a new form of iron-dependent cell death, was formally proposed by Dr. Brent R Stockwell in 2012 (Dixon et al., 2012). From a morphological and molecular point of view, it is characterized by dysmorphic small mitochondria, decreased mitochondrial crests, increased mitochondrial membrane density, ruptured outer mitochondrial membranes, normal nuclear size, and the absence of condense chromatin, caused by the overwhelming accumulation of lipid peroxidation and oxidative disturbance of the intracellular microenvironment (Xie et al., 2016; Li et al., 2020a). Mechanistically, based on previous studies, ferroptosis occurs when the natural defense mechanisms that prevent the accumulation of lipid peroxides are compromised (Stockwell, 2022). Briefly, cysteine is transported into the membrane via the cystine/glutamate reverse transporter (system Xc-), while glutamate is transported out of the membrane. This step can be inhibited easily by sulfasalazine and erastin. System Xc− is composed of two subunits SLC7A11 and SLC3A2 that exchange extracellular cysteine and intracellular glutamate at a ratio that modulates their redox state (Koppula et al., 2018; Liu et al., 2021a). Subsequently, cysteine is reduced in the cystine reduction pathway dependent on glutathione (GSH) or TXNRD1, promoting GSH production (Kuang et al., 2020). Due to its strong reducing properties, GSH serves as a primary cofactor of glutathione peroxidase 4 (GPX4), which removes excess lipid peroxide and promotes the reduction of phospholipid hydrogen peroxides (PLOOHs) to their corresponding alcohols (PLOHs) in cells. Additionally, GSH4 is involved in the production of oxidized glutathione (GSSG) (Maiorino et al., 2018).

**FIGURE 3:**
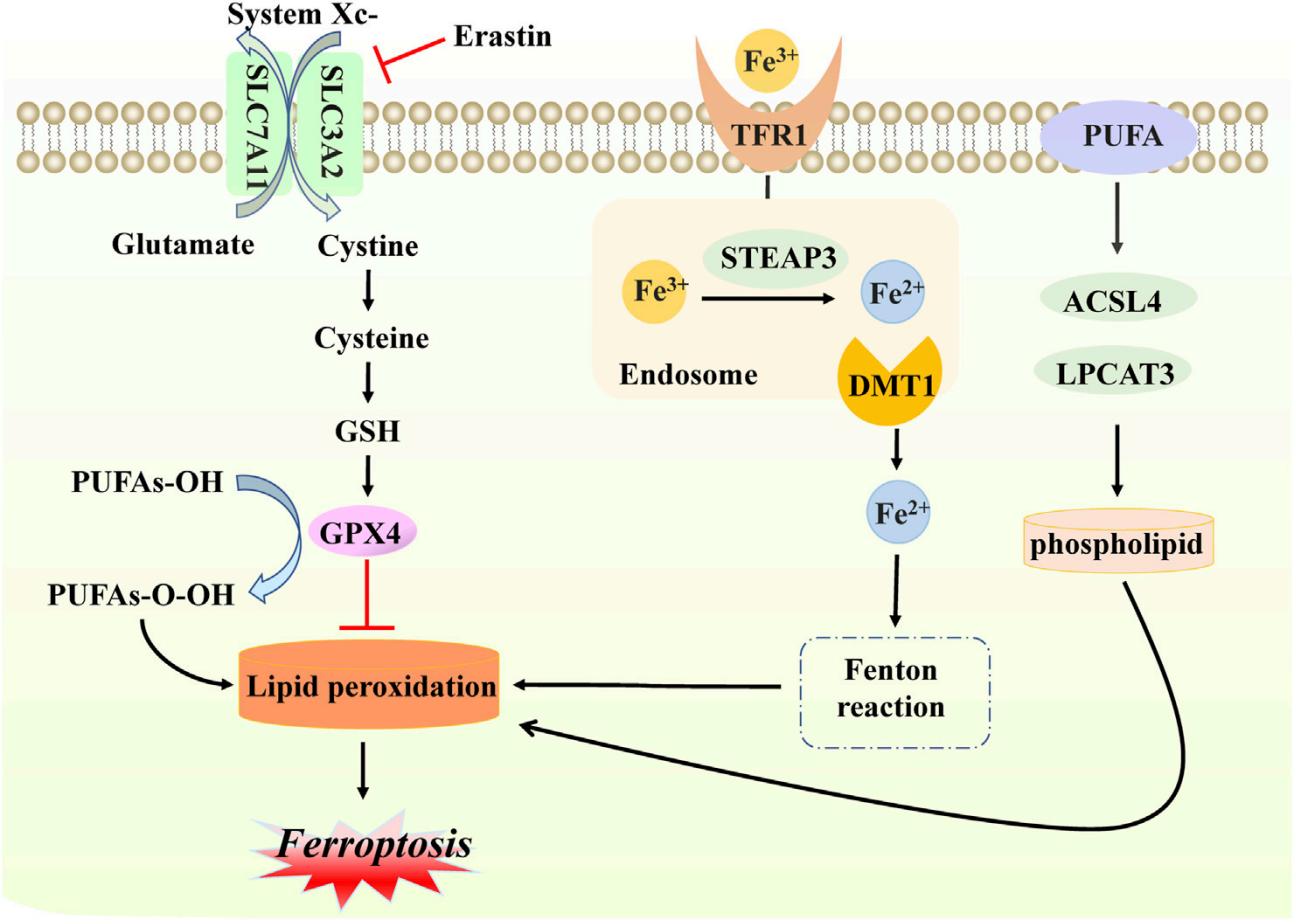
Molecular mechanisms of ferroptosis. In ferroptosis, cystine enters and exits the cell membrane through system Xc− (composed of SLC7A11 and SLC3A2 proteins); this step can be inhibited by erastin. With the accumulation of cystine, GSH levels increase, and GPX4 is activated, inhibiting lipid peroxidation production. When free iron atoms bind to TFR1, they are reduced to ferrous protein STEAP3. Additionally, DMTI ensures that ferrous proteins are present in large quantities in the form of divalent iron, which subsequently initiates lipid peroxidation through the Fenton reaction with hydrogen peroxide in the cells. PUFA also interferes with ACSL4 and LPCAT3, with enzymes involved in the activated biosynthesis of phospholipid, which is necessary for lipid peroxidation. The production of the above lipid peroxidation will eventually cause death in the form of ferroptosis.

One of the key elements of ferroptosis often manifests in the accumulation of iron in cells. Given the presence of a low abundance of ferroportin, responsible for iron efflux, and a high level of transferrin receptor 1 (TFR1), responsible for cellular iron uptake, extracellular ferric iron transports into cells, binding to transferrin, and reduces to ferrous protein through six-transmembrane epithelial antigen of prostate family member 3 (STEAP3) (Masaldan et al., 2018; Chen et al., 2021). Subsequently, ferrous protein can be transported into ferrous iron with the help of mammalian iron transporters, including DMT1 (Cadieux et al., 2012; Song et al., 2021). Reduction to a ferrous state, combined with hydrogen peroxide, is likely to produce a Fenton reaction, which damages cells and tissues through the generation of excessive oxidation of polyunsaturated fatty acids and reactive oxygen species, thereby resulting in ferroptosis (Yanatori and Kishi, 2019). Above all, three main events occur in ferroptosis, namely, the inhibition of the cystine/glutamate transporter system, the increase of lipid peroxidation products, and progressive iron-related lipid ROS accumulation. Additionally, treatment of polyunsaturated fatty acids (PUFAs) is required for the onset of ferroptosis, and its function relies on the activation of enzymes involved in biosynthesis, such as ACSL4 and LPCAT3. These are then attached to acyl-coenzyme A derivatives to produce phosphatidylethanolamine (PE), identified as producing membrane lipids that are prone to peroxidation, ultimately leading to ferroptosis (Jiang et al., 2021b; Gan, 2022) (Figure 3).

### 2.4 NETosis

**FIGURE 4:**
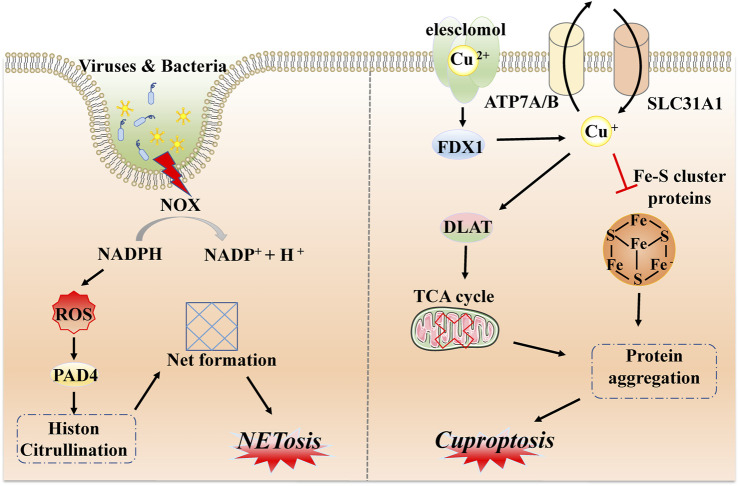
Molecular mechanisms of NETosis and cuproptosis. NETosis is a form of inflammatory response based on the production of NADPH oxidase-mediated ROS via external stimulation, such as by viruses and bacteria, followed by transcription and translation of key protein PAD4 and citrullination with nuclear histones to form network structures via chromatin depolymerization. Cuproptosis is driven by excess copper ions binding to a potent copper ionophore known as elesclomol. Subsequently, a large quantity of divalent copper ions can be reduced to copper ions by FDX4 and applied to induce abnormal oligomerization of thiolated proteins through the thiolated proteins in TCA; in addition, copper ions can also reduce Fe-S cluster protein levels, which together induce proteotoxic stress and eventually lead to cell death.

After the identification of common forms of programmed cell death, such as necroptosis, pyroptosis, and ferroptosis, substantial efforts were made to identify other unfamiliar types of PCD, including NETosis, mitotic catastrophe, parthanatos, and anoikis. NETosis is a form of PCD driven by NET release, which consists of the release of depolymerized chromatin and intracellular granular proteins in response to infection or injury. It is a form of inflammatory cell death of neutrophils, first observed in 2004 (Brinkmann et al., 2004). The unique morphological changes indicate leafy depolymerization of the loss of the nucleus and cytoplasmic granule membranes, contact of chromatin with intracytoplasmic granules, cell membrane damage, and the formation of an extracellular net-like structure. The occurrence of NETosis requires the production of NADPH oxidase-mediated ROS and involves pathogen-activated platelets (e.g., activated by bacteria, fungi, or viruses), interleukin 8, transforming growth factor beta, and other inducers (Remijsen et al., 2011; Brinkmann, 2018). These inducers can promote the transcription and translation of NETosis-crucial proteins, namely, peptidyl arginine deiminase IV (PAD4). Subsequently, PAD4 is transformed from the cytoplasm, translocated to the nucleus, and citrullinated with nuclear histones, which facilitates chromatin depolymerization to form a network structure (Demers et al., 2012; Grayson and Kaplan, 2016; Perdomo et al., 2019). In addition, the release of neutrophil elastase (ELANE), matrix metalloproteinases (MMP), and myeloperoxidase (MPO) from cytoplasmic granules may lead to cytoskeletal disassembly, protein exposition, and extracellular release (Yipp et al., 2012; Bukong et al., 2018). The initiation of NETosis is a dynamic, complex process with different pathways and the formation of nets; the sequences involved in each step need further mechanistic exploration (Figure 4).

### 2.5 Cuproptosis

Similarly to iron, as an essential cofactor in all organisms, the homeostasis of the internal environment in relation to copper is critical for various physiological processes (Ge et al., 2022). Hence, researchers have recently defined a completely new concept, referred to as cuproptosis. This is a non-apoptotic form of PCD caused by direct binding to lipid-acylated components of the tricarboxylic acid cycle (TCA) (Tsvetkov et al., 2022). Changes in morphological features are less reported, but are mostly related to mitochondrial shrinkage and rupture of mitochondrial membranes (Bian et al., 2022). Distinct from all other known types, different copper-binding molecules or potent copper ionophores, such as elesclomol, are required as a first step to increase levels of intracellular copper. Ferredoxin 1 (FDX1), one of the direct targets of elesclomol, encodes a reductase to reduce Cu2+ to Cu+ and regulates protein lipoylation. Moreover, three copper transport proteins (SLC31A1, ATP7A, and ATP7B) also determine copper homeostasis. SLC31A1 is responsible for copper uptake, while ATP7A and ATP7B are responsible for copper transfer out. The mechanism of cell death caused by dysregulation of copper homeostasis is consistent with the mechanism of cell death induced by copper ion carriers (Li et al., 2022b; Tang et al., 2022; Tsvetkov et al., 2022). Because DLAT and DLST are components of the TCA cycle, FDX1 then directs oligomerization of DLAT and DLST, reducing their levels; this implies that cuproptosis targets components of the TCA cycle (Tsvetkov et al., 2022). It has been demonstrated that mitochondrial respiration is associated with copper death; excess copper promotes aggregation of lipoylated protein and reduces iron-sulfur cluster protein; this triggers aggregation and dysregulation of these proteins, blocking the TCA cycle, leading to proteotoxic stress and ultimately cell death (Huang et al., 2022). Briefly, cuproptosis mainly depends on intracellular copper accumulation and the TCA cycle, while FDX1 and proteolipidation are key regulators (Figure 4).

## 3 Links between programmed cell death regulation and carcinogenesis

### 3.1 Links between regulation of necrosis and carcinogenesis

As previously mentioned, the process of activation of necroptosis is crucial to prevent uncontrolled inflammatory responses and resulting pathological conditions, such as the occurrence of cancer (Najafov et al., 2017; Xie et al., 2020). Necroptosis activation has been reported to correlate well with invasive phenotypes and poor prognosis in HNSCC; indeed, some necroptosis-related proteins, including RIPK1, RIPK3, and MLKL, have been found to be higher in mice with OSCC, suggesting that necroptosis is a crucial event in oral injuries (Wei et al., 2022). Since the release of DAMPs is one of the key features of necroptosis, it has been elucidated that necroptotic IL-1β activates the NF-κB pathway and further leads to increased migration and invasion in HNSCC, based on findings in two related cell lines. This result indicated that necroptosis might be a potential tumor promoter in HNSCC (Li et al., 2020b). Another study has demonstrated that HNSCC harbors the most frequent genomic amplifications of the Fas-associated death domain (FADD), which is ascribed to the fact that RIPK1 undergoes deubiquitination events. The authors identified FADD knockdown as inhibiting HNSCC, displaying amplification and increased expression, associated with the occurrence of necroptosis *in vitro* (Eytan et al., 2016). Consequently, targeting of the necroptotic pathway seems to be a potential therapeutic route with HNSCC oncogenic effects.

### 3.2 Links between regulation of pyroptosis and carcinogenesis

Recently, an increasing number of studies have proposed pyroptosis regulation as a crucial event in the evolution of HNSCC. GSDME, as the major executive protein of pyroptosis, has been reported to be strongly expressed in OSCC in a study using a human OSCC tissue microarray (Wang et al., 2022). Wu et al. constructed a scoring system to assess pyroptosis dysfunction on the basis of integrated TCGA genomic data in each participant in a cohort of HNSCC patients (Wu et al., 2022b). The low-scoring group had a more comprehensive tumor mutational load than the high-scoring group, particularly in TP53 and TTN, indicating that pyroptosis features in the tumor microenvironment. Kaplan–Meier survival curves were used to construct gene clusters of HNSCC patients based on the expression of pyroptosis mRNA, as well as to compare immune cell infiltration in the high- and low-scoring pyroptosis groups. Low pyroptosis scores were associated with a stronger response to immune activation, implying that pyroptosis is associated with immunotherapy in HNSCC (Deng et al., 2022). Differentially expressed pyroptosis-related genes might have an impact on prognosis in HNSCC. Zhu et al. concluded that NLRP3 can be identified as a potential predictor based on relative expression outcomes of tumor samples paired with adjacent normal tissue samples in the context of HNSCC (Zhu et al., 2022). Given that NLRP3 is one of the most widely studied receptor proteins at the beginning of pyroptosis, the findings also indicated that NLRP3 offers potential value in HNSCC development. Many methodological approaches, such as RT-qPCR assays and immunohistochemical analyses, can be exploited to demonstrate the relationship between pyroptosis and HNSCC. Additionally, an investigation performed by Bottino et al. sheds light on pyroptosis, indicating that it could serve as a promising mechanism for regulation of the roles of tumor-derived extracellular vesicles (Bottino et al., 2021). It is possible to downregulate gene expression of NLRP3, pro-IL-1β, and pro-caspase-1 proteins that influence the priming phase of the inflammatory response in the HNSCC microenvironment (Zhang et al., 2020; Shen et al., 2022). In summary, the characteristics of pyroptosis in HNSCC are mainly related to prognosis and immunotherapy.

### 3.3 Links between regulation of ferroptosis and carcinogenesis

In recent years, it has been reported that deregulation of ferroptosis is associated with numerous cancers, including HNSCC. Indeed, this form of regulated cell death can play a fundamental role in suppressing tumor growth. Many studies have demonstrated the importance of a key regulator of ferroptosis, solute carrier family 7 member 11 (SLC7A11), which transports extracellular cystine into cells for glutathione biosynthesis triggered by lipid peroxidation (Xie et al., 2016; Koppula et al., 2021). Shi et al. have suggested that both SLC7A11 and GPX4 are highly expressed in the progression of the HNSCC cell line, regulating ferroptosis by positively affecting B cells, CD8^+^ T cells, and CD4^+^ T cells (Shi et al., 2021). Additionally, through reduction in ferroptosis, the overexpression of SLC7A11, regulated by NRF2 nuclear, has been found to be positively modulated by lymph node metastasis in esophageal squamous cell carcinoma tissues (Feng et al., 2021). To explore the application of combination ferroptosis regulation within a tumor environment, a comprehensive multi-omics analysis of three distinct ferroptosis patterns was conducted in patients with OSCC; these were found to be linked to low gene copy number burden, high levels of immune checkpoint expression, and prognosis (Gu et al., 2021). Another study has also validated the prognostic value of ferroptosis regulation in OSCC. The authors identified 25 ferroptosis-related differentially expressed lncRNAs, revealing a high-risk lncRNA signature associated with poor prognosis in HNSCC (Tang et al., 2021). Furthermore, advanced detection methods involving single-cell transcriptomics have revealed that essential ferroptosis regulators contribute to the development of HNSCC, including muscular contraction and humoral immunological responses, and play a crucial role in immune infiltration (Liu et al., 2022). Therapies associated with HNSCC can be expected to focus on the regulation of ferroptosis cell death regulators, such as GPX4 and SLC7A11, and HNSCC prognosis.

### 3.4 Links between regulation of NETosis and cuproptosis and carcinogenesis

As the first line of defense at the site of infection, neutrophils play an important role in the innate immune system. Tumor cells can cause NETosis *in vivo* and *in vitro* (Tanaka et al., 2014; Huang et al., 2015). Accumulating evidence from multiple studies suggests that NETosis appears to be associated with tumorigenesis, progression, and metastasis, indicating that NETosis has a direct effect on tumor cell proliferation via protease or activation signaling (Sangaletti et al., 2012; Tripodo et al., 2017). Of note, the relationship between OSCC and NETosis can be observed in the formation of NETs in a coculture of neutrophils with CAL-27 cell lines, accompanied by expression of the PI3K/Akt/PBK pathway (Garley et al., 2020). Overall, Decker suggests that elevated NETosis in the blood can be used as a valid biomarker for the detection of early HNSCC progression and prevention of tumor metastasis (Decker et al., 2019).

Cuproptosis has been recognized as a novel form of regulatory cell death, and has been confirmed to promote the occurrence and development of tumors. Based on exploration through bioinformatics, researchers have established that cuproptosis-related lncRNA has an impact on prognosis in HNSCC (Li et al., 2022c; Yang et al., 2022). By comparing data on overall survival, risk score distribution, and survival status by group, we elucidated the relationship between cuproptosis and the immune microenvironment in HNSCC, highlighting the fact that cuproptosis metabolism may be a possible predictive biomarker for HNSCC treatment (Zhang et al., 2022). Additionally, the results suggested that OSCC cell metastasis is closely associated with cuproptosis with high expression of AFOC-DEGs (Li et al., 2022a).

## 4 Effects of natural products associated with programmed cell death on carcinogenesis

Recently, therapeutic approaches aiming to identify and characterize representative regulators of programmed necroptosis have represented an emerging field in the treatment of HNSCC. Natural plants are considered to be an invaluable resource in targeting these pathways and appropriating them for therapeutic benefit.

### 4.1 Effects of natural products associated with necroptosis on carcinogenesis

Considering that natural products are fully present in many plants and foods with preventive and curative properties, representing an inexhaustible source of active molecules, these substances need to be further explored for anticancer drug discovery in the future. A predominant type of naphthoquinone, namely, shikonin, a compound derived from the Chinese plant *Lithospermum erythrorhizon*, has been found to exert inhibitory effects on tumor growth. One study has reported an increase in reactive oxygen species (ROS) production and observed dose-dependent upregulation of RIPK1, RIPK3, and MLKL, leading to the induction of necroptosis. With a strong inhibitory effect on 5–8F cells, the shikonin group in this study exhibited transparent cytoplasm and incomplete plasma membrane, indicating that shikonin may exhibit promising properties in the treatment of nasopharyngeal carcinoma (Liu et al., 2019a). Similarly, paeoniflorigenone, a plant extract from the family Paeoniaceae, inhibits necroptosis in human YD-10B HNSCC cells. This extract has been used for medicinal purposes against inflammatory diseases, including cancer. Paeoniflorigenone leads to the dephosphorylation of key necroptotic proteins (RIP and MLKL) in YD-10B cells, significantly suppressing cell migration and invasion (Park et al., 2022). Certain food compounds, including capsaicin, that restore the susceptibility of cancer cells to standard chemotherapeutic treatments have been described many times in the literature. One study has ultimately suggested that necroptosis might be a major cause of the inhibition of the cell viability of oral squamous carcinoma cells resulting from co-application of capsaicin with four conventional anticancer agents (Huang et al., 2021b). In summary, a final goal in this domain is to assess these active compounds to define their molecular roles in HNSCC treatment and subsequently investigate them clinically for the prevention or treatment of incurable diseases based on the mechanisms underlying necroptosis.

### 4.2 Effects of natural products associated with pyroptosis on carcinogenesis

Triptolide (TPL), a trioxide diterpene, is one of the main active components of Tripterygium wilfordii Hook F (TwHF), which exhibits potent anticancer effects in multiple cancers (Jiang et al., 2021a; Deng et al., 2021). TPL has been found to induce pyroptosis-like cell death in a nasopharynx cancer cell line, with a decrease of cell viability, the release of IL-1β cytokine, cytoplasmic swelling, and membrane rupture, but no apoptosis. Lysis of GSDME and activation of the caspase-3 pathway on the mitochondria were observed in this experiment, underlining the essential role of decreased tumorigenicity in this type of cell death (Cai et al., 2021). Anthocyanins, which are glycoside derivatives, are polyphenol compounds that are widely employed in cancer prevention at various stages (Lin et al., 2017). Researchers have also demonstrated that anthocyanin, in combination with caspase-1 inhibitors, can induce pyroptosis while suppressing OSCC carcinogenic activities. Their results also suggest that NLRP3, caspase-1, and IL-1β are essential components in the investigation of pyroptosis (Yue et al., 2019). Because studies of this type of cell death have not been sufficiently powerful, novel active compounds are required to distinguish the contributions of caspase-1/3/11 to the pyroptotic signaling pathway in HNSCC.

### 4.3 Effects of natural products associated with ferroptosis on carcinogenesis

It is well known that artemisinin engages in outstanding antimalarial activity; however, artemisinin is also renowned for its special biological interest in the domain of antitumor therapy, such as in renal cancer, breast cancer, and HNSCC (Liu et al., 2019b; Li et al., 2021; Zheng et al., 2022). Artesunate has been found to exhibit a specific cytotoxic effect on HNSCC cells through the upregulation of lipid reactive oxygen species (ROS) generation and the downregulation of cellular glutathione (GSH) levels, both of which are crucial mediators of ferroptosis (Dixon et al., 2012; Roh et al., 2017). Collectively, this effect may be suboptimal in dihydroartemisinin because of the activation of both ferroptosis and apoptosis pathways. The administration of DHA could arrest cells in an iron-dependent manner, and possibly by means of arresting cell circles in the G2/M phase (Lin et al., 2016). Both *in vitro* and vivo models have demonstrated that dihydroartemisinin is a ferroptosis inducer, thereby increasing cellular iron-free levels and increasing sensitivity to cancers (Chen et al., 2020). Very recently, it has been shown that a widely available tetracyclic triterpenoid molecule found in natural products, namely, cucurbitacin B (CuB), triggers ferroptosis. This compound is one of the most abundant members of the Trichosanthes kirilowii Maximowicz family, with a wide range of pharmacological activities, such as strong anti-inflammatory, antipyretic, and anticancer activities (Chan et al., 2010; Dakeng et al., 2012). Furthermore, it has been reported that CuB is associated with intracellular accumulation of iron ions and GSH depletion in human nasopharyngeal carcinoma cells, resulting in the downregulation of lipid peroxidation and GPX4 expression, and eventually leading to ferroptosis (Huang et al., 2021a). Importantly, natural compounds regulate ferroptosis for HNSCC progression by targeting the GSH/GPX4 pathway, lipid metabolism, and iron metabolism.

## 5 Conclusion and future perspectives

Over the last few decades, the majority of the attention in this field has been focused on the ways in which apoptosis and autophagy modes are activated in cancer cells; nevertheless, further in-depth studies of the complexity of cancer cells may indicate that they are probably not able to avoid multiple different kinds of cell death simultaneously. In presenting this review, we have paved the way for the more likely development of the other relevant modes of PCD that have been reported in the context of HNSCC (Table 1).

**TABLE 1:** Current reports concerning hallmarks of major types of RCD.

Type	Inducer	Morphological features	Biochemical features	Major regulators
Necroptosis	Caspase-8 inhabitation	damage of plasma membrane; lysis of endoplasmic reticulum; swelling of mitochondrial; release of intracellular contents	Activation of RIPK1/RIPK3/MLKL; cytosolic necrosome formation	Positive: RIPK1, RIPK3, and MLKL
Pyroptosis	DAMPs&PAMPs; LPS	fewer swelling cells in mitochondrial, extensive cytoplasmic vacuolization, pore formation and rupture on plasma membrane; release of intracellular contents	Activation of CASP1; Activation of CASP4/5/11; pyroptosome formation; GSDMD-N–induced pore formation; IL1β/IL-18 release	Positive: CASP1, CASP4/5/11, and GSDMD
Ferroptosis	Excess iron; Erastin	dysmorphic small mitochondria, decreased mitochondrial crests, increased mitochondrial membrane density, ruptured outer mitochondrial membranes	Iron accumulation; lipid peroxidation; caspase-independent	Positive: ACSL4, LPCAT3 Negative: SLC7A11,SLC3A2 GPX4
Netosis	pathogens (e.g., bacteria, fungi, viruses)	loss of the nucleus and cytoplasmic granule membranes, contact of chromatin with intracytoplasmic granules, cell membrane damage and form a extracellular net-like structure	Neutrophil activation; production of NADPH oxidase-mediated ROS; PAD4-mediated Citrullination	Positive: PAD4, MPO, MMP, ELANE

Among these modes of cell death, inducers are mostly related to external factors, such as in the cases of ferroptosis and copoptosis, both of which are associated with the accumulation of relevant ions, while pyroptosis and necroptosis are influenced by stimulated inflammatory responses, with the exception of necroptosis occurring in line with caspase-8 inactivation.

All of these patterns involve multiple organelles that are regulated in terms of cellular morphology. Necroptosis, pyroptosis, ferroptosis, and copoptosis are generally associated with the mitochondria, although necroptosis is primarily associated with chromatin depolymerization, and the production of net structures is also a representative marker in NETosis. Research has shown that necroptosis and pyroptosis are primarily characterized by mitochondrial swelling, plasma membrane cleavage, and the release of intracellular contents, whereas ferroptosis and copoptosis are mostly characterized by the rupture of the outer mitochondrial membrane and wrinkling of the shape, with the nucleus remaining relatively normal in size and with an intact cytoplasmic membrane. In contrast, pyroptosis is distinguished by pore development and substantial cytoplasmic vacuolization. Regarding biochemical features, necroptosis and pyroptosis are closely related to the formation of the necrosome and pyroptosome, and ROS are generated in necroptosis, ferroptosis, and NETosis; in addition, ferroptosis and copoptosis are regulated by caspase-independent pathways.

Based on distinctive molecular mechanisms and major regulators, one promising therapeutic avenue that has emerged in potentially overcoming necroptosis, pyroptosis, and ferroptosis resistance in tumor cells is the application of natural products; some of these, as described in our review, can be used as prognosis markers for neoplastic diseases. Nevertheless, it also should be noted that the development of a specific PCD inhibitor remains a significant therapeutic need. For instance, these may include inhibitors of RIPK1/3 targeting the necroptotic machinery, such as the natural product nigratine (also known as 6E11), a flavanone derivative, which is regarded as an inhibitor of RIPK1 kinase targeting the necroptotic machinery and has been found to effectively protect aortic endothelial cells from cold hypoxia-reoxygenation injury (Delehouzé et al., 2017; Delehouzé et al., 2022).

Pyroptosis is driven by caspase-1-dependent or -independent pathways and by NLRP3, which is associated with a multi-protein complex referred to as the pyroptosome. Preliminary evidence shows that distinct checkpoints are set in place to mediate pyroptosis between the caspase-1 pathway and pyroptosome production. Luteoloside, a flavonoid isolated from *Gentiana macrophylla*, has shown promise for hepatocellular carcinoma invasion and metastasis as a caspase-1 and NLRP3 inflammasome inhibitor (Fan et al., 2014).

Moreover, a distinct checkpoint is set in place to mediate ferroptosis through the ubiquitination of GPX4. DMOCPTL, a derivative of the natural product parthenolide, has been found to exert an anticancer effect in inhibiting breast tumor growth and to prolong mouse lifespan by directly binding to GPX4 protein (Ding et al., 2021). On the whole, therapeutic strategies targeting small-molecule inhibitors could be a viable approach for defense against HNSCC.

At one time, open surgery was considered to be a risky treatment option for HNSCC, as it often resulted in disfiguring side effects and even death due to complications. Therefore, alternative therapeutic approaches, such as pharmaceutical therapy and radiation therapy, have been developed to target tumor cells and induce their safe demise. At present, however, with the deepening of our understanding of unique molecular regulators and mechanisms for each type of PCD, many questions remain unanswered. Possible considerations for the cancer therapy field are: which PCD mode should be preferred to induce distinct tumors? Should these modes be employed simultaneously or sequentially? What is the interplay between these different modes of PCD? Although much is known regarding the biological activity involved in each type of PCD, the molecular events upstream and downstream of major regulators remain largely obscure.

In summary, it is valuable for the development of targeted therapies to define more reliable regulators and molecules to better pinpoint critical therapeutic windows. More in-depth clinical studies and further explorations of the factors associated with different PCD modes are needed to develop more accurate and individualized methods for the diagnosis and treatment of HNSCC. With a better understanding of the complex mechanisms involved in PCD and associated potential therapies, we anticipate a breakthrough in novel drug discovery for the prevention of HNSCC in the future.
